# A new species of *Odorrana* (Anura, Ranidae) from the limestone karst forest of northern Vietnam

**DOI:** 10.3897/zookeys.1280.192981

**Published:** 2026-05-22

**Authors:** Cuong The Pham, Chung Van Hoang, Mai Hong Thi Nguyen, Truong Quang Nguyen, Hai Ngoc Ngo, Dzung Trung Le, Thomas Ziegler

**Affiliations:** 1 Institute of Biology, Vietnam Academy of Science and Technology, 18 Hoang Quoc Viet Road, Hanoi 10072, Vietnam Institute of Zoology, University of Cologne Cologne Germany; 2 Graduate University of Science and Technology, Vietnam Academy of Science and Technology, 18 Hoang Quoc Viet, Cau Giay, Hanoi 10072, Vietnam Graduate University of Science and Technology, Vietnam Academy of Science and Technology Hanoi Vietnam; 3 Institute of Theoretical and Applied Research, Duy Tan University, 100000 Hanoi, Vietnam Institute of Biology, Vietnam Academy of Science and Technology Hanoi Vietnam; 4 School of Medicine and Pharmacy, Duy Tan University, 550000 Da Nang, Vietnam Faculty of Biology, Thai Nguyen University of Education Thai Nguyen Vietnam; 5 Faculty of Biology, Thai Nguyen University of Education, Thai Nguyen, Vietnam Institute of Theoretical and Applied Research, Duy Tan University Hanoi Vietnam; 6 Cologne Zoo, Riehler Straße 173, 50735, Cologne, Germany School of Medicine and Pharmacy, Duy Tan University Da Nang Vietnam; 7 Institute of Zoology, University of Cologne, Zülpicher Straße 47b, 50674, Cologne, Germany Cologne Zoo Cologne Germany

**Keywords:** Karst forest, molecular phylogeny, *Odorrana nagao* sp. nov., taxonomy, Tuyen Quang Province

## Abstract

A new species of *Odorrana* is described from the limestone karst forest in northern Vietnam based on morphological differences and molecular divergence. Morphologically, *Odorrana nagao***sp. nov**. is distinguishable from its congeners by the following combination of characteristics: size medium (SVL 37.4 mm in male, 47.1–54.7 mm in females); head longer than wide; tympanum distinct, round, ~50% of the diameter of eye; vomerine teeth present; males without external vocal sac; nuptial pad present on finger I in males; webbing formula I0–1II0–1III0–11/3IV11/3–1/4V; tibio-tarsal articulation reaching the tip of snout when hindlimb adpressed along body; dorsolateral conical spines present; dorsolateral fold present; dorsum moss-green with brown mottling; ventral surface cream with dark brown mottling. Phylogenetic analyses of mitochondrial 16S rRNA and COI gene sequences supported the new species as a sister taxon to an unresolved clade consisting of *Odorrana calciphila*, *O. concelata*, *O. feii*, *O. liboensis* and *O. lipuensis* (UFB = 98, PP = 1). The uncorrected p-distances of 16S rRNA and COI genes between the new species and its closest congeners were 2.33% and 6.33%, respectively.

## Introduction

Karst landscapes across Asia, extending from China to western Melanesia, represent an important center of biodiversity and are characterized by a high level of endemism (Clements et al. 2006; Grismer et al. 2021). The genus *Odorrana* Fei, Ye & Huang, 1990, is among the most diverse amphibian groups with 70 recognized odorous frogs so far, more than 20 of which were described in the last ten years (Frost 2026). The *Odorrana* taxa are widely distributed in Asia, from northeastern India and southern China eastwards to Japan, throughout Indochina and southwards to Sumatra and Borneo (Pham et al. 2025; Frost 2026). Most of them are associated with mountain streams, except for *Odorrana wuchuanensis* (Xu, 1983), *O. lipuensis* Mo, Chen, Wu, Zhang & Zhou, 2015; *O. mutschmanni* Pham, Nguyen, Le, Bonkowski & Ziegler, 2016; *O. liboensis* Luo, Wang, Xiao, Wang & Zhou, 2021; *O. concelata* Wang, Zeng & Lin, 2022; *O. calciphila* Song, Qi, Wang, Liu & Wang, 2025 and *O. feii* Li, Mu, Jing, Liu, Cheng & Wang, 2025; which are restricted to limestone karst landscapes (Fei et al. 2009, 2012; Liu and Wang 2014; Mo et al. 2015; Pham et al. 2016a, 2016b; Luo et al. 2021; Lin et al. 2022; Li et al. 2025; Song et al. 2025).

Based on morphological characteristics, the karst-associated *Odorrana* taxa can be assigned to two distinct groups, one consisting of *Odorrana wuchuanensis* and *O. mutschmanni*, and the other comprising *O. lipuensis*, *O. liboensis*, *O. concelata*, *O. calciphila* and *O. feii* (Pham et al. 2016b; Lin et al. 2022; Li et al. 2025; Song et al. 2025). Phylogenetic studies further revealed a sister relationship between *O. wuchuanensis* and *O. mutschmanni* within the *O. margaretae* group, and *O. lipuensis*, *O. liboensis*, *O. concelata*, *O. calciphila* and *O. feii* together forming a monophyletic group, designated as the *O. lipuensis* group (Pham et al. 2016b; Luo et al. 2021; Lin et al. 2022; Li et al. 2025; Song et al. 2025). These demonstrate a distinct divergence within the karst adapted groups of *Odorrana* and underscore the complex evolutionary processes associated with karst landscapes in the genus.

During our fieldwork in Tuyen Quang Province, northern Vietnam, five specimens of *Odorrana* were collected in the karst forest of Cham Chu Nature Reserve. These specimens were assigned to the *O. lipuensis* group based on the following morphological characteristics: adult females slightly larger than adult males; vocal sac absent in males; discs of fingers III and IV prominently enlarged, transverse oval, the disc of finger IV the widest; finger I without lateroventral grooves; dorsal skin relatively smooth, with tiny flat granules forming homogeneous worm-like texture; and dorsum with mixed irregular moss-green speckles and brown mottling (Lin et al. 2022; Li et al. 2025; Song et al. 2025). However, the newly discovered population of *Odorrana* from Cham Chu NR differs from other species in the *O. lipuensis* group in several morphological characteristics as well as genetic divergence. We herein describe this taxon as a new species.

## Materials and methods

### Sampling

A field survey was conducted in the limestone karst forest of Cham Chu Nature Reserve, Tuyen Quang Province, Vietnam in April 2019 by C.T. Pham, C.V. Hoang, D.T. Le, A.M. Luong, T.Q. Phan (hereafter Pham et al.). Geographic coordinates and elevations were obtained by using a Garmin GPSMAP 78S satellite communicator. After live photography, frogs were anaesthetized and euthanized in a closed vessel with a piece of cotton wool containing ethyl acetate (Simmons 2002), fixed in 80% ethanol for five hours, and then later transferred to 70% ethanol for permanent storage. Tissue samples were preserved separately in 70% ethanol prior to fixation. Sex was determined by direct observation of calling males in life or by gonadal dissection. All specimens were deposited in the collection of the Institute of Biology (**IB**), Hanoi, Vietnam.

### Molecular data and phylogenetic analyses

We sequenced two new samples of *Odorrana* collected in Cham Chu Nature Reserve, Tuyen Quang Province, Vietnam. Morphologically, the frog specimens from Cham Chu Nature Reserve were considered representatives of the *O. lipuensis* species group. We used the 16S rRNA and COI available sequences of 51 samples of *Odorrana* from GenBank. Outgroup polarity was provided by sequences of *Amolops ricketti* (Li et al. 2025) (Suppl. material 1).

Tissue samples were extracted using PureLink™ RNA Micro Scale Kit (Thermo Fisher Scientific, Lithuania), following the manufacturer’s instructions. Total DNA was amplified using a PCR Applied Biosystems machine. PCR total volume was 25 μl, consisting of 12 μl of Mastermix, 6 μl of water, 1 μl of each primer at a concentration of 10 pmol/μl, and 5 μl of DNA. Primers used in the PCR and sequencing were as follows: the primer pair, LR N 13398 (5’-CGCCTGTTTACCAAAAACAT- 3’; forward), LR J 12887 (5’-CCGGTCTGAACTCAGATCACGT-3’; reverse) (Simon et al. 1994) was used to amplify a fragment of the mitochondrial 16S rRNA gene; the primer pair Chmf4 (5’-TYTCWACWAAYCAYAAAGAYATCGG-3’) and Chmr4 (5’ ACYTCRGGRTGRCCRAARAATCA-3’) (Che et al. 2012) was used to amplify a fragment of the COI gene. PCR products were sent to Apical Scientific company for sequencing (https://apicalscientific.com). The obtained sequences were deposited in GenBank under accession numbers PZ332369–PZ332370 and PZ334602–PZ334603 (Suppl. material 1).

Chromas Pro software (Technelysium Pty Ltd., Tewantin, Australia) was used to edit the sequences. Each gene was then initially aligned separately using ClustalW (Thompson et al. 1997) with default settings for complete alignment. Pairwise comparisons of uncorrected sequence divergence (p distance) were calculated using MEGA11 (Tamura et al. 2021). Data were then analyzed using maximum likelihood (ML) as implemented in IQ-TREE (Nguyen et al. 2015) and Bayesian inference (BI) as implemented in MrBayes v. 3.2 (Ronquist et al. 2012). For the BI and ML analysis, the combined 16S rRNA and COI dataset was partitioned separately and COI was also partitioned into three parts by codon positions (first, second and third) to be used in the multiple-model BI and ML analyses. The best partition scheme and evolutionary model for each partition were selected using Kakusan 4 (Tanabe 2011) and jmodeltest v. 2.1.10 (Darriba et al. 2012). For BI analyses, the Markov chain Monte Carlo (MCMC) algorithms were run for 10^7^ generations with three heated and one cold, starting from random trees and samples one out of every 1,000 generations. The burn-in and convergence diagnostic were assessed using Tracer v. 1.7.1 to confirm Effective Sample Size (ESS) > 200 for all parameters (Rambaut et al. 2018). The cut-off point for the burn-in function was set to 25% of the total number of trees generated. For ML analyses, 10,000 ultrafast bootstrap replications (UFB) were run (Hoang et al. 2018). We considered Bayesian posterior probability (PP) ≥ 0.95 and UFB ≥ 95 as strong support for a clade (Ronquist et al. 2012; Hoang et al. 2018).

### Morphological characters

Measurements were taken with a digital caliper to the nearest 0.1 mm. The following abbreviations were used (following Pham et al. 2016b; Li et al. 2025; Song et al. 2025):

**SVL** snout-vent length (from tip of snout to cloaca);

**HL** head length (a parallel line with the vertebral column from posterior margin of mandible to tip of snout);

**HW** maximum head width (at rictus);

**RL** rostral length (from anterior corner of orbit to tip of snout);

**ED** eye diameter (from the anterior corner to posterior corner of the eye);

**NS** distance from nostril to tip of snout;

**EN** distance from anterior corner of orbit to nostril;

**IND** internarial distance (distance between nostrils);

**IOD** interorbital distance (minimum between upper eyelids);

**UEW** maximum width of upper eyelid;

**DAE** distance between anterior margins of orbits;

**DFE** distance between posterior margins of orbits;

**MN** distance from posterior margin of mandible to nostril;

**MFE** distance from posterior margin of mandible to anterior margin of orbit;

**MBE** distance from posterior margin of mandible to posterior margin of eye;

**TD** tympanum diameter (from anterior margin to posterior margin of the tympanum);

**TYE** distance from anterior margin of tympanum to posterior corner of orbit;

**UAL** upper arm length (from axilla to elbow);

**RAD** radiulna length (from the flexed elbow to the wrist);

**HND** hand length (from the wrist to the tip of finger III);

**FL1–4** finger length I–IV (from inner to outer);

**NPL** nuptial pad length – finger I;

**fd3** width of discs of fingers III;

**FeL** femur length (from vent to knee);

**TbL** tibia length (from knee to tarsus);

**TbW** maximum tibia width;

**FoL** foot length (from tarsus to tip of fourth toe);

**TL1–5** toe length I–V;

**td4** width of discs of toe IV;

**IMT** inner metatarsal tubercle length.

For webbing formula, we followed Glaw and Vences (2007). Sex was determined by the presence of nuptial pads and based on gonadal inspection.

Morphological data used for comparison were obtained from the literature (Suppl. material 2).

### Statistical analyses

For the statistical analyses, the newly discovered population was compared to its closest relatives based on the phylogeny of *Odorrana*. Raw morphological data used for the analyses were obtained from the new specimens collected in Cham Chu Nature Reserve, and from 44 specimens representing the five other *Odorrana* species (including six *O. calciphila*, eight *O. concelata*, 13 *O. liboensis*, nine *O. lipuensis*, and eight *O. feii*), available from previous studies (Li et al. 2025; Pham et al. 2025; Song et al. 2025). Their raw data are presented in Table 1.

**Table 1.** Morphological characteristics (mm) of *Odorrana nagao* sp. nov. and its five closest congeners of *Odorrana*, along with comparison results of ANOVA and TukeyHSD post hoc tests. P-values indicate significant differences between the new species and each of the five congeners (***: < 0.0001; **: 0.001–0.01; *: < 0.05). Asterisks in *Odorrana nagao*. sp. nov. indicate differences in the overall ANOVA test among all species for each characteristic.

Characteristics	*O. calciphila*	*O. concelata*	*O. feii*	*O. liboensis*	*O. lipuensis*	*Odorrana nagao* sp. nov.
SVL	37.90–45.70 (42.42 ± 1.31)	33.90–46.00 (38.04 ± 1.55) (**)	37.00–52.20 (44.26 ± 2.44)	47.80–61.40 (54.48 ± 1.01)	40.70–57.90 (48.81 ± 2.03)	37.40–54.70 (49.24 ± 3.25) (***)
HW	12.50–15.10 (13.90 ± 0.47) (***)	10.60–14.60 (11.88 ± 0.48) (***)	12.10–17.10 (14.66 ± 0.79) (***)	15.50–21.10 (18.40 ± 0.36) (***)	13.20–20.50 (16.39 ± 0.93)	12.20–18.10 (16.38 ± 1.09) (***)
HL	13.70–16.60 (15.12 ± 0.52) (***)	11.80–15.30 (13.16 ± 0.45) (***)	13.40–16.90 (15.01 ± 0.54) (***)	17.80–23.80 (20.60 ± 0.39) (*)	14.60–21.00 (17.26 ± 0.79) (***)	14.30–21.10 (19.16 ± 1.26) (***)
RL	5.40–6.90 (6.20 ± 0.23) (***)	5.10–6.70 (5.47 ± 0.19) (***)	5.80–7.80 (6.81 ± 0.27) (**)	7.00–9.70 (8.57 ± 0.20) (***)	5.60–8.40 (7.26 ± 0.38)	5.70–8.30 (7.52 ± 0.48) (***)
IND	3.70–4.40 (4.02 ± 0.12) (***)	3.20–4.20 (3.51 ± 0.13) (***)	4.20–5.60 (4.89 ± 0.19)	4.90–6.80 (5.73 ± 0.16) (**)	3.40–5.60 (4.67 ± 0.23)	3.50–5.60 (4.96 ± 0.39) (***)
IOD	2.80–4.20 (3.58 ± 0.22) (***)	3.00–4.40 (3.34 ± 0.17) (***)	4.00–5.40 (4.71 ± 0.21)	4.20–7.90 (5.90 ± 0.38)	3.30–5.60 (4.42 ± 0.24)	3.60–6.20 (5.26 ± 0.44) (***)
ED	4.70–5.50 (5.08 ± 0.17) (**)	3.80–6.00 (4.39 ± 0.24) (***)	5.10–6.20 (5.61 ± 0.13)	5.50–8.10 (6.88 ± 0.21)	4.30–7.40 (5.41 ± 0.36) (**)	5.20–7.10 (6.46 ± 0.33) (***)
TD	3.10–3.90 (3.60 ± 0.11)	3.20–3.40 (3.30 ± 0.03)	3.40–5.00 (4.26 ± 0.19) (***)	4.00–5.90 (4.82 ± 0.15) (***)	3.20–5.10 (4.07 ± 0.22) (*)	2.80–3.90 (3.52 ± 0.19) (***)
HND	10.50–13.20 (12.10 ± 0.41) (***)	9.20–12.90 (10.70 ± 0.51) (***)	10.60–15.40 (13.28 ± 0.72) (***)	14.40–17.40 (15.76 ± 0.24)	10.30–17.20 (13.58 ± 0.74) (***)	11.30–16.70 (15.18 ± 1.00) (***)
RAD	8.20–10.40 (9.57 ± 0.32) (***)	7.10–9.80 (8.31 ± 0.33) (***)	8.30–11.40 (9.66 ± 0.45) (***)	11.10–14.60 (12.55 ± 0.32)	7.70–12.20 (10.38 ± 0.63) (***)	9.20–13.70 (12.32 ± 0.82) (***)
FTL	27.50–32.90 (31.12 ± 0.83) (***)	24.00–32.20 (26.90 ± 1.06) (***)	27.90–37.90 (32.99 ± 1.47) (***)	37.00–48.50 (41.29 ± 0.91)	30.90–39.20 (35.17 ± 1.13) (***)	30.90–42.70 (39.54 ± 2.18) (***)
TIB	19.10–23.80 (22.30 ± 0.73) (***)	17.30–23.30 (19.35 ± 0.75) (***)	19.80–27.50 (23.86 ± 1.04) (***)	26.50–41.50 (31.36 ± 1.42)	21.60–28.90 (25.78 ± 0.86) (***)	21.40–39.90 (30.10 ± 2.95) (***)

All statistical analyses were conducted in R v. 4.5.2 (R Core Team 2025). To remove the effects of allometry in the morphometric characters, raw data of the six *Odorrana* species were normalized, using the following equation: Xadj = log(X) – ß[log(SVL)-log(SVL_mean_)], where Xadj = adjusted value; X = measured value; ß = unstandardized regression coefficient for each species and SVL_mean_ = overall average SVL of all species (Thorpe 1975, 1983; Turan 1999; Lleonart et al. 2000; Chan and Grismer 2022). Boxplots were generated for eleven size–corrected morphometric characteristics of six selected *Odorrana* species (i.e., HL, HW, RL, IND, IOD, ED, TD, HND, RAD, FTL and TIB), excepting for SVL with raw data. One-way analysis of variance (ANOVA) for an overall difference, and followed by Tukey’s HSD posthoc-test for pairwise differences were applied in each characteristics among the six species. Morphospatial relationships among species were visualized using principal component analysis (PCA) based on the eleven size–corrected morphometric characteristics, using packages of “factoextra” (Kassambara and Mundt 2020) and “FactoMineR” in R (Le et al. 2008). A PERMANOVA analysis calculating an Euclidean (dis)similarity matrix using 9,999 permutations, from the vegan package 2.5–3 in R (Oksanen et al. 2020) was applied to determine if the centroid group clusters of each species from the PCA were statistically different, based on the individual scores of the first four PC dimensions (PC1 – PC4). Pairwise PERMANOVA comparisons were further performed to assess differences in the PC dimensions between species pairs.

## Results

### Molecular phylogenetic analysis

**Figure 1.**
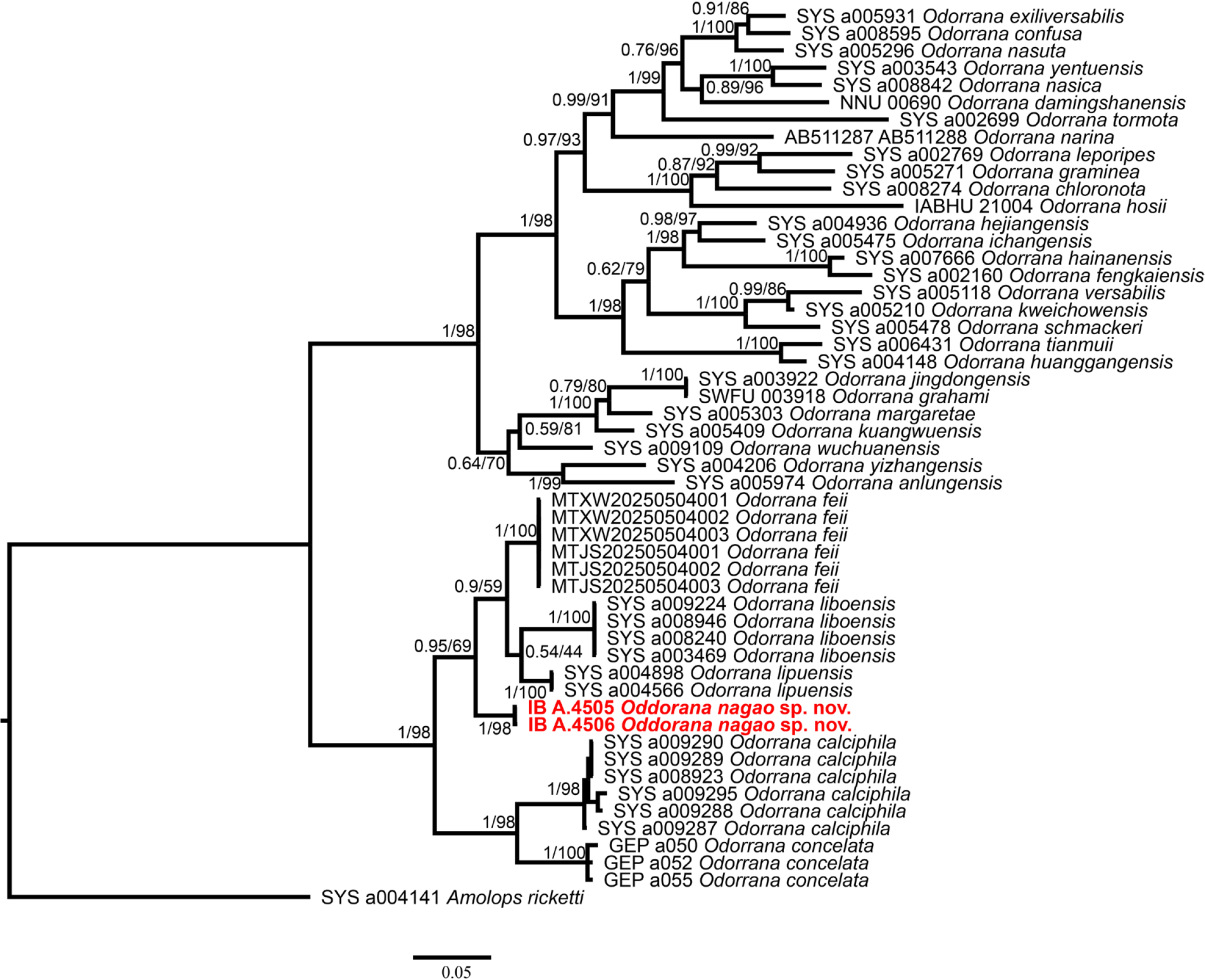
Bayesian phylogram based on a partial 16S and COI mitochondrial fragment. Numbers above and below branches are PP/UFB bootstrap values and Bayesian posterior probabilities (> 50%), respectively.

The combined matrix of 16S rRNA and COI contained 1,116 aligned characters (16S: 528 and COI: 588), of which 713 were constant, 364 parsimony­-informative and 403 variable character parsimony-uninformative. The new form of *Odorrana* in Cham Chu Nature Reserve, was recovered as monophyletic in an unresolved clade consisting of *O. calciphila*, *O. concelata*, *O. feii*, *O. liboensis* and *O. lipuensis* (UFB = 98, PP = 1) (Fig. 1). However, the new form was divergent from other members in the *O. lipuensis* group by at least 2.33% and 6.33% based on 16S and COI genes, respectively (Tables 2, 3).

**Table 2.** Uncorrected pairwise genetic distance (%) between members of the *Odorrana lipuensis* group estimated from 16S sequences.

	Species	1	2	3	4	5	6
**1**	*Odorrana nagao* sp. nov.	**0.00**					
**2**	*Odorrana concelata*	5.21	**0.00–0.19**				
**3**	*Odorrana feii*	2.33	6.03–6.24	**0.00**			
**4**	*Odorrana lipuensis*	2.34	6.04–6.24	2.14	**0.00**		
**5**	*Odorrana liboensis*	3.75	6.26–6.47	3.13	3.75	**0.00**	
**6**	*Odorrana calciphila*	5.00–6.50	3.54–4.79	5.20–6.70	5.83–7.35	6.27–7.35	**0.00–1.55**

**Table 3.** Uncorrected pairwise genetic distance (%) between members of the *Odorrana lipuensis* group estimated from COI sequences.

	Species	1	2	3	4	5
**1**	*Odorrana nagao* sp. nov.	**0.00–0.17**				
**2**	*Odorrana feii*	6.33–6.52	**0.00**			
**3**	*Odorrana lipuensis*	7.48–7.67	4.60	**0.00**		
**4**	*Odorrana liboensis*	9.49	6.87	5.72	**0.00**	
**5**	*Odorrana calciphila*	11.61–11.82	14.10–14.32	12.82–13.03	15.26–15.49	**0.00–0.68**

In the following, based on distinct genetic divergence in concert with diagnostic morphological differences compared to congeners, we describe the newly discovered population of *Odorrana* from Cham Chu Nature Reserve, Tuyen Quang Province, Vietnam as a new species.

### Statistical analyses

**Figure 2.**
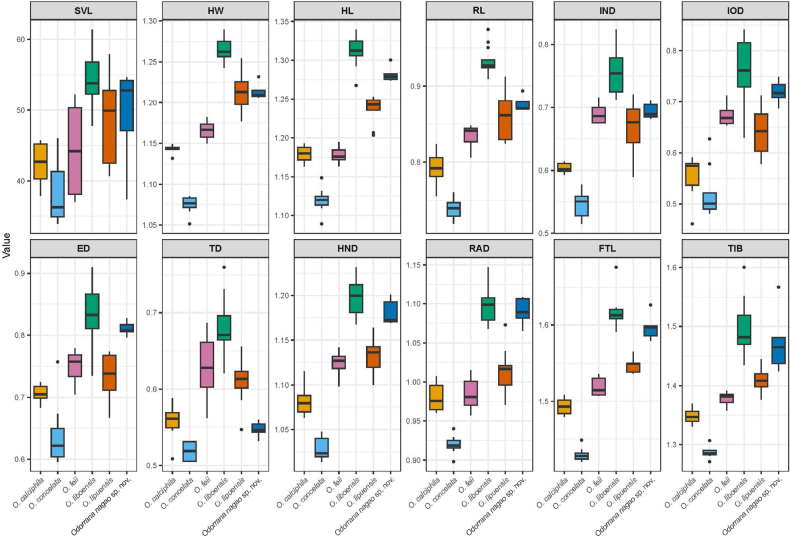
Boxplots of raw snout-vent length (SVL) and eleven size-corrected morphological characteristics of *Odorrana nagao* sp. nov. and five closely related phylogenetic lineages of *Odorrana*.

The ANOVA tests revealed all six *Odorrana* species are significantly different in the snout-vent length (SVL) and 11 size-corrected characteristics (all tests with P-values < 0.05), and TukeyHSD post hoc tests showed that the *Odorrana* population from Cham Chu Nature Reserve (namely, *Odorrana nagao* sp. nov.) is significantly different from *O. calciphila* in HW, HL, RL, IND, IOD, ED, HND, RAD, FTL and TIB; from *O. concelata* in SVL, HW, HL, RL, IND, IOD, ED, HND, RAD, FTL and TIB; from *O. feii* in HW, HL, RL, TD, HND, RAD, FTL and TIB; from *O. liboensis* in HW, HL, RL, IND and TD; from *O. lipuensis* in HL, ED, TD, HND, RAD, FTL and TIB (Table 1; Fig. 2).

**Figure 3.**
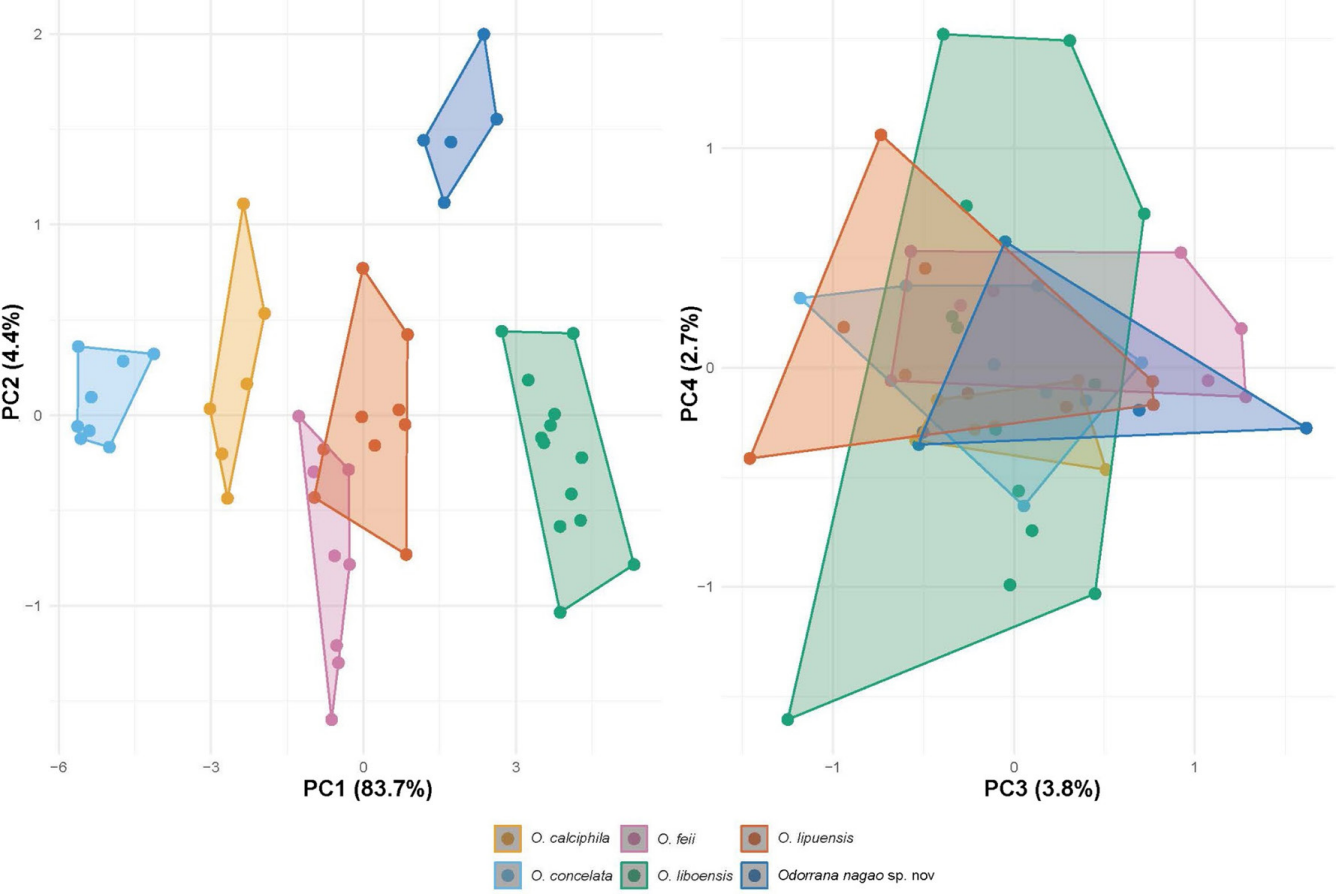
Principal component analysis (PCA) of *Odorrana nagao* sp. nov., and five closely phylogenetic lineages of *Odorrana* (*O. calciphila*, *O. concelata*, *O. liboensis*, *O. lipuensis* and *O. feii*).

In the PCA analysis, the first four principal components (PC1 – PC4) explained 83.7%, 4.4%, 3.8% and 2.7% of the total variance, respectively, accounting for 94.6% cumulatively. The clustering pattern in the PCA scatterplot based on size-corrected data showed that the samples of *Odorrana nagao* sp. nov. from Cham Chu Nature Reserve form a distinct cluster that non-overlaps with those of other species along the ordination of the first two components (Fig. 3). Furthermore, the PERMANOVA test demonstrates that all six selected species of *Odorrana* are statistically different in the morphospace of combined first four PCs (R^2^ = 0.89; F = 75.08; P < 0.001). Additionally, pairwise PERMANOVA tests revealed a significant difference between the new species and each of the remaining *Odorrana* species in the PCs: *Odorrana nagao* sp. nov. vs *O. calciphila* (R^2^ = 0.59; F = 17.03; P < 0.001), vs *O. concelata* (R^2^ = 0.93; F = 157; P < 0.001), vs *O. feii* (R^2^ = 0.71; F = 27.76; P < 0.05), vs *O. liboensis* (R^2^ = 0.48; F = 14.99; P < 0.001), vs *O. lipuensis* (R^2^ = 0.51; F = 12.52; P < 0.001).

### Taxonomic account

#### Odorrana nagao sp. nov.

Taxon classificationAnimaliaAnuraRanidae

FA4DCB78-DCBD-507C-B31E-B01A4E265E47

https://zoobank.org/EFEC6E09-0E75-4E08-A2F8-49415FE99C69

**Figure 4.**
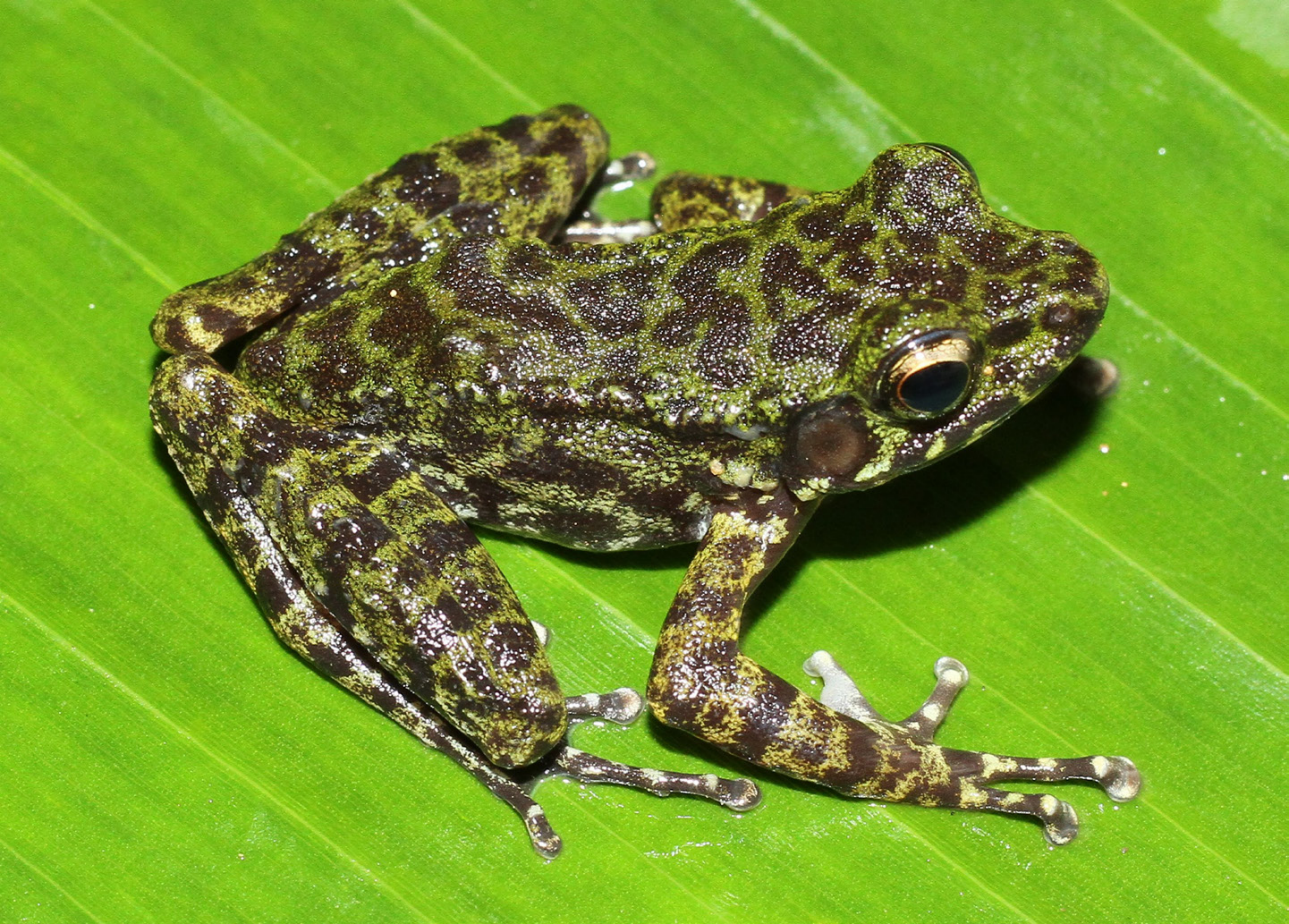
Dorsolateral view of *Odorrana nagao* sp. nov., holotype (IB A.4505, male) in life.

**Figure 5.**
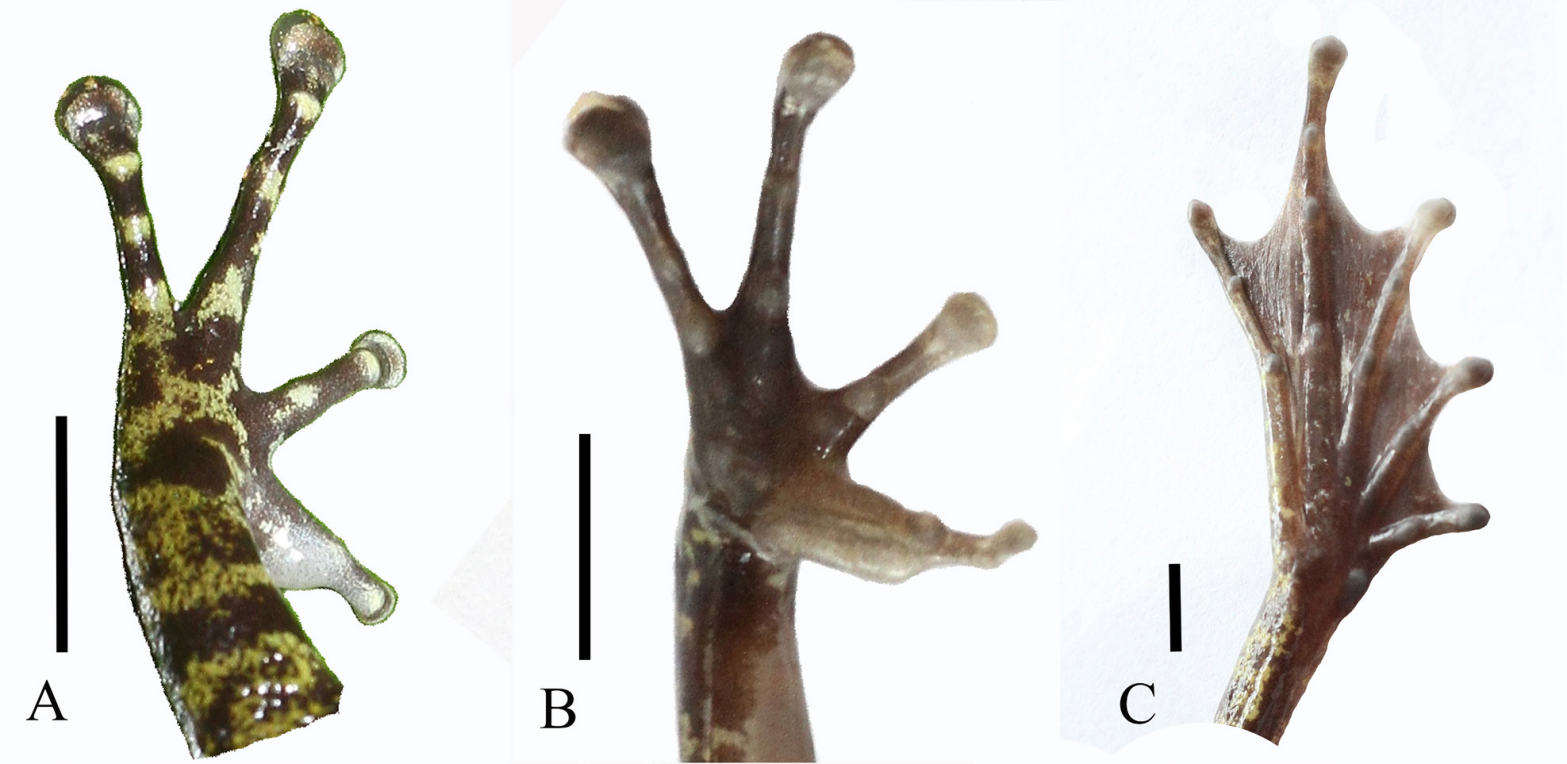
*Odorrana nagao* sp. nov., holotype (IB A.4505, male). **A**. Dorsal view of left hand; **B**. Underside of right hand; **C**. Underside of right foot. Scale bars: 5 mm

**Figure 6.**
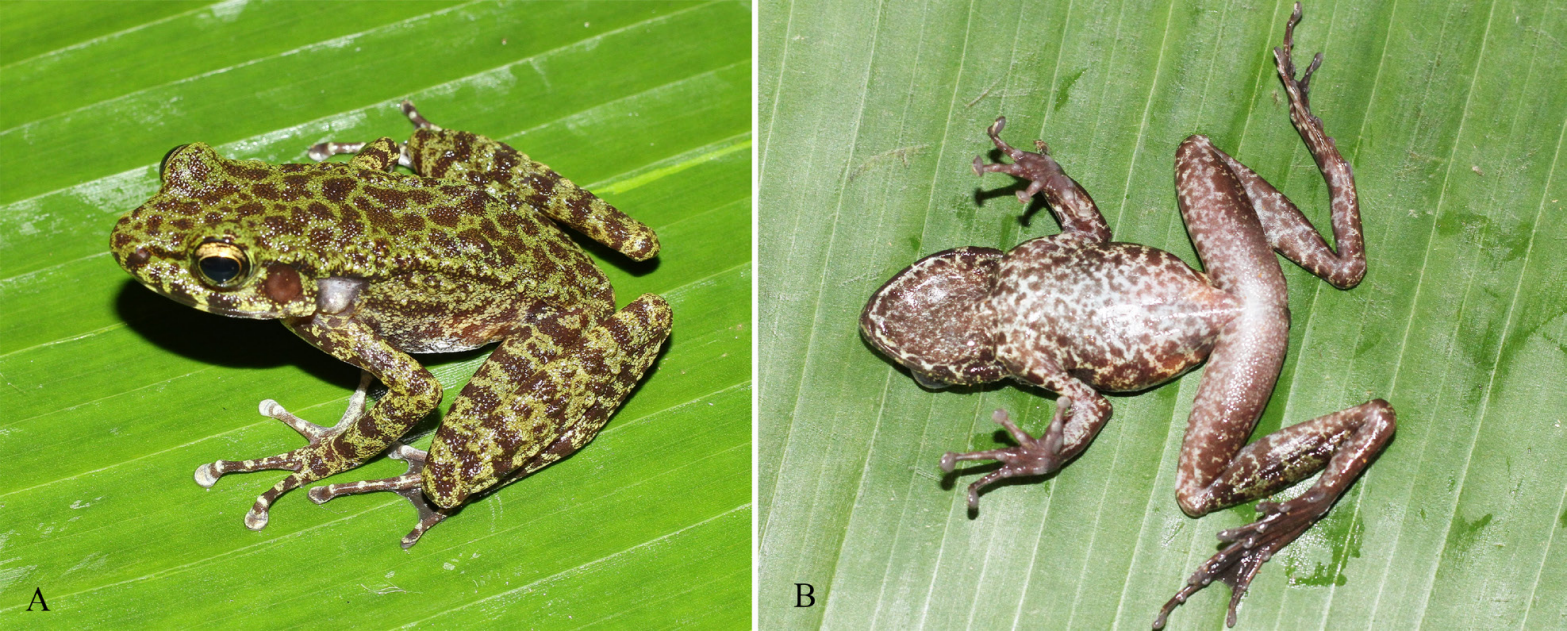
*Odorrana nagao* sp. nov., paratype (IB A.4506, female) in life. **A**. Dorsolateral view; **B**. Ventral view.

Figs 4, 5, 6, Table 4

##### Type material.

***Holotype***. • IB A.4505, adult male, collected by Pham et al. on 12 April 2019 in the karst forest near Cao Duong Village (22°17.380'N, 104°59.427'E, at an elevation of 641 m a.s.l.), Cham Chu Nature Reserve, Tuyen Quang Province, Vietnam. ***Paratypes***. • IB A.4506, adult female (the same data as the holotype); • IB A.4507–A.4509, adult females, collected by Pham et al. on 13 April 2019 in the karst forest near Cao Duong Village (22°18.036'N, 104°59.447'E, at an elevation of 773 m a.s.l.), Cham Chu Nature Reserve, Tuyen Quang Province, Vietnam.

##### Diagnosis.

The new species is assigned to the *Odorrana lipuensis* group on the basis of the following morphological characteristics: adult females slightly larger than adult males; vocal sac absent in males; discs of fingers III and IV prominently enlarged, transverse oval, the disc of finger IV the widest; finger I without lateroventral grooves; dorsal skin relatively smooth, with tiny flat granules forming homogeneous worm-like texture; and dorsum with mixed irregular moss-green speckles and brown mottling (Lin et al. 2022; Li et al. 2025; Song et al. 2025). *Odorrana nagao* sp. nov. is distinguishable from its congeners by a combination of the following morphological characters: (1) size medium (SVL 37.4 mm in male, 47.1–54.7 mm in females); (2) head longer than wide; (3) tympanum distinct, round, ~50% of the diameter of eye; (4) vomerine teeth present; (5) male without external vocal sac; (6) nuptial pad present on finger I in male; (7) webbing formula I0–1II0–1III0–11/3IV11/3–1/4V; (8) tibio-tarsal articulation reaching the tip of snout when hindlimb adpressed along body; (9) dorsolateral conical spines present; (10) dorsolateral fold present; (11) dorsum moss-green with brown mottling; (12) ventral surface cream with dark brown mottling.

##### Description of holotype.

Adult male, size medium (SVL 37.4 mm); head longer than wide (HL 14.3 mm, HW 12.2 mm); snout obtusely round in dorsal view, projecting beyond lower jaw, round in profile; nostril lateral, closer to snout tip than eye (NS 2.3 mm, EN 3.4 mm); canthus distinct; pupil horizontally oval; loreal region slightly concave and oblique; rostral length greater than eye diameter (RL 5.7 mm, ED 5.2 mm); interorbital distance wider than internarial distance and upper eyelid (IOD 3.6 mm, IND 3.4 mm, UEW 2.9 mm); tympanum distinct, round, smaller than eye diameter (TD/ED 0.54); vomerine teeth present, on two oblique ridges; tongue cordiform, deeply notched posteriorly; external vocal sac absent.

Forelimbs slender; fingers slender, relative finger lengths II < I < IV < III; tips of fingers II, III, and IV expanded into discs with lateroventral grooves, discs of fingers III and IV prominently enlarged, transverse oval, the disc of finger IV the widest, tip of finger I without lateroventral grooves; fingers free of webbing; subarticular tubercles round, formula 1, 1, 2, 2; inner metatarsal tubercle elongate; outer metatarsal tubercle small; nuptial pad present on finger I.

Hindlimbs long; thigh shorter than tibia (FeL 18.4 mm, TbL 21.4 mm); tibia ~5× longer than wide (TbL/TbW 5.2), tips of toes expanded into discs with lateroventral grooves; relative toe lengths I < II < III < V < IV; webbing well developed, formula I0–1II0–1III0–11/3IV11/3–1/4V; subarticular tubercles present, formula 1, 1, 2, 3, 2; inner metatarsal tubercle oval; outer metatarsal tubercle absent; tibio-tarsal articulation reaching the tip of snout when hindlimb adpressed along body.

##### Skin texture in life.

Dorsal surface of head and body smooth; flank and dorsal surface of fore and hind limbs with small tubercles; tiny spinules present on anterior and posterior edge of tympanum; supratympanic fold weakly developed; dorsolateral conical spines present; dorsolateral fold present; throat, chest, belly and ventral surface of thigh smooth.

##### Coloration in life.

Iris black, surrounded by a golden network; dorsum and upper part of flank moss-green with brown mottling; dorsal surface of fore and hind limbs moss-green with dark brown cross bars; upper lip with dark brown bars; ventral surface of limb slightly pink; throat, chest, and belly cream with dark brown mottling; toe webbing dark brown.

##### Variation.

Male smaller than females; male with nuptial pads present on finger I (absent in females), and females having creamy yellow eggs, without black poles. Measurements and morphological characters of the type series are given in Table 4.

**Table 4.** Measurements (in mm) and proportions of the type series of *Odorrana nagao* sp. nov. (H = holotype, P = paratype, SD = standard deviation, M = male, F = female; for other abbreviations see Materials and methods).

Voucher	IB A.4505	IB A.4506	IB A.4507	IB A.4508	IB A.4509	Min.–Max.	Mean ± SD
Sex	M	F	F	F	F	(*N* = 4)	(*N* = 4)
Type status	H	P	P	P	P		
SVL	37.4	54.2	54.7	52.8	47.1	47.1–54.7	52.2 ± 3.5
AG	16.2	24.7	25.4	23.9	19.8	19.8–25.4	23.5 ± 2.5
HW	12.2	18.1	17.9	17.4	16.3	16.3–18.1	17.4 ± 0.8
HL	14.3	21.1	20.9	20.4	19.1	19.1–21.1	20.1 ± 0.9
MN	12.5	17.3	17.7	17.1	16.6	16.3–18.1	17.1 ± 0.5
MFE	9.5	12.7	12.8	12.3	12.2	12.2–12.8	12.5 ± 0.3
MBE	5.2	7.4	7.5	7.2	7.4	7.2–7.5	7.3 ± 0.1
RL	5.7	8.3	8.2	7.9	7.5	7.5–8.3	8.0 ± 0.4
ED	5.2	6.9	7.1	6.6	6.5	6.5–7.1	6.8 ± 0.3
NS	2.5	4.0	3.7	3.9	3.3	3.3–4.0	3.7 ± 0.3
EN	3.2	4.3	4.5	4.3	4.1	4.1–4.5	4.3 ± 0.2
TD	2.8	3.9	4.0	3.6	3.5	3.5–4.0	3.8 ± 0.2
TYE	1.2	1.9	2.8	2.2	2.1	1.9–2.8	2.3 ± 0.4
UEW	2.9	4.2	4.4	4.1	4.1	4.1–4.4	4.2 ± 0.1
IOD	3.6	5.5	6.2	5.7	5.3	5.3–6.2	5.7 ± 0.4
IND	3.4	5.4	5.5	5.6	4.8	4.8–5.6	5.3 ± 0.4
DAE	6.5	9.5	9.8	9.4	8.7	8.7–9.8	9.4 ± 0.5
DPE	10.2	14.1	14.3	14.5	13.1	13.1–14.6	14.0 ± 0.6
UAL	7.8	12.1	12.3	12.4	9.8	9.8–12.4	11.7 ± 1.2
RAD	9.2	12.9	13.5	13.7	12.3	12.3–13.7	13.1 ± 0.6
HND	11.3	16.4	16.3	16.7	15.2	15.2–16.7	16.2 ± 0.7
HAL	20.5	29.3	29.8	30.4	27.5	27.5–30.4	29.3 ± 1.3
NPL	3.7	–	–	–	–	–	–
fd3	1.6	2.1	2.4	2.3	1.9	1.9–2.4	2.2 ± 0.2
FeL	18.4	25.8	24.9	27.1	26.3	24.9–27.1	26.0 ± 0.9
TbL	21.4	30.5	29.9	30.9	28.8	28.8–30.9	30.0 ± 0.9
TbW	4.1	5.9	6.4	5.7	5.2	5.2–6.4	5.8 ± 0.5
FoL	30.9	42.7	41.3	42.0	40.8	40.8–42.7	41.7 ± 0.8
td4	1.2	1.6	1.9	1.7	1.5	1.5–1.9	1.7 ± 0.2
HL/SVL	0.38	0.39	0.38	0.39	0.41	0.38–0.41	0.39 ± 0.01
HW/SVL	0.33	0.33	0.33	0.33	0.35	0.33–0.35	0.33 ± 0.01
HL/HW	1.17	1.17	1.16	1.17	1.18	1.16–1.18	1.17 ± 0.01
TD/ED	0.54	0.57	0.56	0.55	0.54	0.54–0.57	0.55 ± 0.01
ED/RL	0.91	0.83	0.87	0.84	0.87	0.83–0.87	0.85 ± 0.02
RL/SVL	0.15	0.15	0.15	0.15	0.16	0.15–0.16	0.15 ± 0.01
NS/EN	0.78	0.93	0.82	0.91	0.80	0.80–0.93	0.87 ± 0.06
IOD/UEW	1.24	1.31	1.41	1.39	1.29	1.29–1.41	1.35 ± 0.06
IOD/IND	1.06	1.02	1.13	1.02	1.10	1.02–1.13	1.07 ± 0.06
UAL/SVL	0.21	0.22	0.22	0.23	0.21	0.21–0.23	0.22 ± 0.01
HAL/SVL	0.55	0.54	0.54	0.58	0.58	0.54–0.58	0.56 ± 0.02
fd3/TD	0.57	0.54	0.60	0.64	0.54	0.54–0.64	0.58 ± 0.05
FeL/SVL	0.49	0.48	0.46	0.51	0.56	0.46–0.51	0.50 ± 0.04
FoL/SVL	0.83	0.79	0.76	0.80	0.87	0.76–0.87	0.80 ± 0.05
TbL/SVL	0.57	0.56	0.55	0.59	0.61	0.55–0.61	0.57 ± 0.03
TbL/TbW	5.22	5.17	4.67	5.42	5.54	4.67–5.42	5.20 ± 0.4
td4/TD	0.43	0.41	0.48	0.47	0.43	0.41–0.48	0.45 ± 0.03

##### Etymology.

The new species is named in honor of the Nagao Natural Environment Foundation, Japan for the support of biodiversity research and nature conservation in Vietnam. For the common names, we suggest Nagao Odorous Frog (English), Ếch đá na-gao (Vietnamese).

##### Distribution.

*Odorrana nagao* sp. nov. is currently known only from the type locality in Cham Chu Nature Reserve, Tuyen Quang Province, Vietnam.

##### Ecological notes.

**Figure 7.**
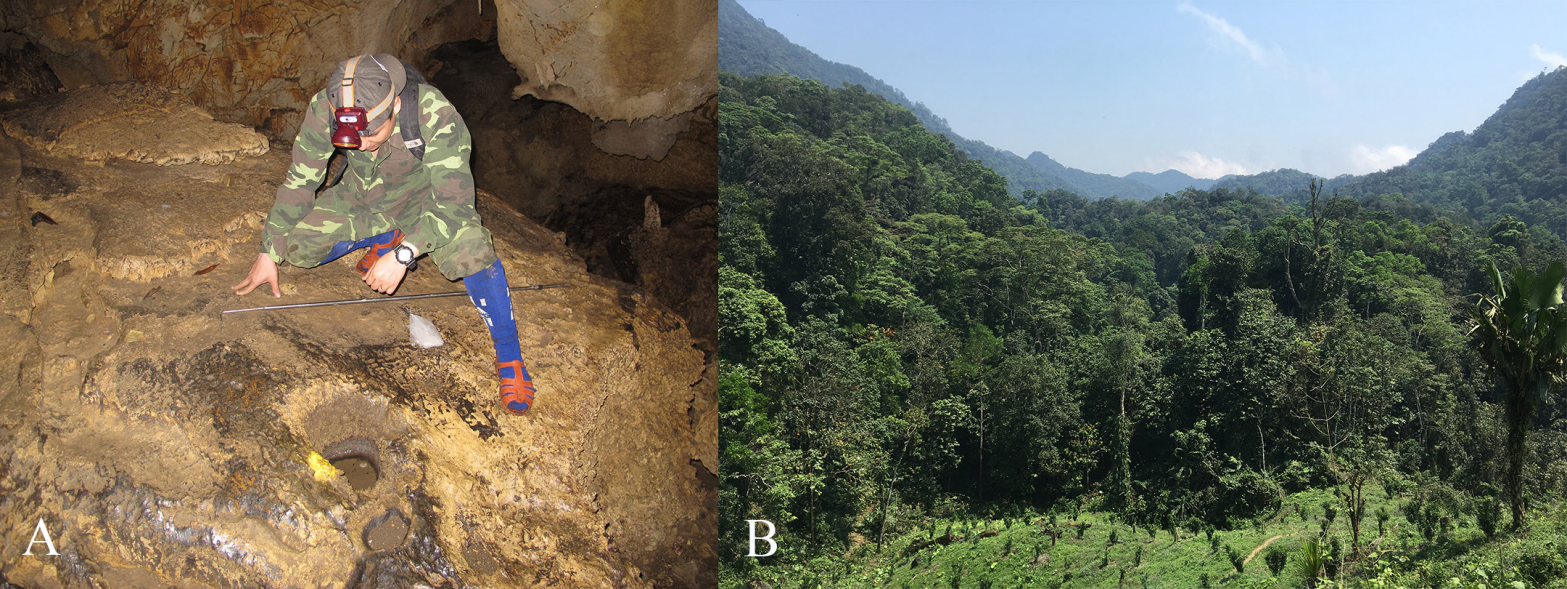
Natural habitat of *Odorrana nagao* sp. nov. in Cham Chu Nature Reserve, Tuyen Quang Province, Viet Nam: **A**. Microhabitat and **B**. Limestone karst forest habitat.

Specimens were found at night between 19:30 and 23:00, on rocks inside caves or near cave entrances, at elevations between 641 and 773 m a.s.l (Fig. 7A). The surrounding habitat is the secondary karst forest, composed of medium and small hardwoods, shrubs, and vines (Fig. 7B). Air temperature was recorded at 23.4–28.5 °C, and relative humidity at 62–86%. Other amphibian species found at the site were *Duttaphrynus melanostictus* (Schneider, 1799), *Microhyla butleri* Boulenger, 1900, *Fejervarya limnocharis* (Gravenhorst, 1829), *Kurixalus hainanus* (Zhao, Wang & Shi, 2005), *Polypedates mutus* (Smith, 1940), and *Rhacophorus orlovi* Ziegler & Köhler, 2001.

##### Morphological comparisons.

Morphological data used for comparisons were obtained from the literature (Suppl. material 2). Based on phylogenetic analysis *Odorrana nagao* sp. nov. is placed within the *O. lipuensis* group. However, *Odorrana nagao* sp. nov. can be morphologically distinguished from its congeners by the following characteristics (Table 5).

**Table 5.** Diagnostic characters separating *Odorrana nagao* sp. nov. from its related species.

Morphological character	*Odorrana nagao* sp. nov.	*O. calciphila*	*O. concelata*	*O. feii*	*O. liboensis*	*O. lipuensis*
SVL in adult males (in mm)	37.4	37.9	33.9–36.8	37.0–39.0	43.7–53.8	40.7–49.8
SVL in adult females (in mm)	47.1–54.7	39.9–45.7	41.3–46.0	49.4–52.2	48.8–58.2	49.9–60.1
Relative finger length	II < I	I < II	II ≤ I	I < II	I < II	II ≤ I
Nuptial pad	Finger I	Fingers I, II, III; nuptial pads on fingers I and II connected	Fingers I, II, III; nuptial pads on fingers I and II not connected	Absent	Finger I	Finger I
Dorsolateral conical spines	Dense	Dense, sparse or absent	Dense or sparse	Sparse or absent	Sparse or absent	Dense
Tibiotarsal articulation (when stretched)	Reaching snout	Exceeding nostril	Reaching nostril	Reaching nostril	Between eye and nostril	Reaching nostril
Dorsolateral fold	Present in both sexes	Prominent and swollen in adult females and absent in adult male	Absent in both sexes	Present in adult females and absent in adult males	Present in adult females and present or absent in adult males	Absent in both sexes
Band on the dorsal surface of hind-limb	Clear	Clear	Clear	Clear	Unclear	Clear

*Odorrana nagao* sp. nov. differs from *O. calciphila* by having a larger body size in females (SVL 47.1–54.7 mm vs 39.9–45.7 mm), dorsolateral fold present in male (vs absent), relative finger length II < I (vs I < II); nuptial pad present on finger I (vs nuptial pads present on fingers I, II, and III), different webbing on toes (webbing formula I0–1II0–1III0–11/3IV11/3–1/4V vs I0–1/2II0–1III1/2–1IV11/2–1/2V), tibiotarsal articulation reaching the tip of snout (vs exceeding nostril);

From *O. concelata* by having a larger body size in females (SVL 47.1–54.7 mm vs 41.4–46.0 mm); a smaller ratio of TD/ED (0.54 in male and 0.55 in females vs 0.82 in males and 0.75 in females), dorsolateral fold present (vs absent), nuptial pad present on finger I (vs nuptial pads present on fingers I, II and III); tibiotarsal articulation reaching the tip of snout (vs tibiotarsal articulation reaches to nostril);

From *O. feii* by having a smaller ratio of TD/ED (0.54 in male and 0.55 in females vs 0.71 in males, 0.79 in females), dorsolateral conical spines dense (vs sparse or absent), dorsolateral fold present in male (vs absent), relative finger length II < I (vs I < II), nuptial pad present on finger I (vs absent), tibiotarsal articulation reaching the tip of snout (vs reaching nostril);

From *O. liboensis* by having a smaller body size (SVL 37.4 mm in male and 47.1–54.7 mm in females vs 45.2–49.9 mm in males and 55.8–61.4 mm in females), a greater ratio of ED/TD in females (1.77–1.86 vs 1.10–1.35), dorsolateral conical spine dense (vs sparse or absent), dorsolateral fold present in male (vs absent), relative finger length II < I (vs I < II), different webbing on toes (webbing formula I0–1II0–1III0–11/3IV11/3–1/4V vs I0–1/4II0–1/2III0–1IV1–0V), tibiotarsal articulation reaching the tip of snout (vs reaching between eye and nostril);

From *O. lipuensis* by having a smaller body size in males (SVL 37.4 mm vs 40.7–49.8 mm), a greater ratio of (ED/TD 1.86 in male and 1.77–1.86 in females vs 1.23–1.50 in males and 1.14–1.44 in females), dorsolateral fold present (vs absent), different webbing on toes (webbing formula I0–1II0–1III0–11/3IV11/3–1/4V vs I0–1/4II0–1/2III0–3/4IV3/4–0V), tibiotarsal articulation reaching the tip of snout (vs reaching nostril), and inner metatarsal tubercle oval on hindlimbs (vs elongate).

*Odorrana nagao* sp. nov. differs from other congeners by the absence of the vocal sac in males (vs present in *Odorrana absita*, *O. amamiensis*, *O. anlungensis*, *O. aureola*, *O. bacboensis*, *O. banaorum*, *O. bolavensis*, *O. cangyuanensis*, *O. chapaensis*, *O. chloronota*, *O. confusa*, *O. damingshanensis*, *O. dulongensis*, *O. exiliversabilis*, *O. fengkaiensis*, *O. geminata*, *O. gigatympana*, *O. grahami*, *O. graminea*, *O. hainanensis*, *O. heatwolei*, *O. hejiangensis*, *O. hosii*, *O. huanggangensis*, *O. ichangensis*, *O. indeprensa*, *O. ishikawae*, *O. jingdongensis*, *O. junlianensis*, *O. khalam*, *O. kweichowensis*, *O. leporipes*, *O. livida*, *O. lungshengensis*, *O. macrotympana*, *O. monjerai*, *O. morafkai*, *O. nanjiangensis*, *O. nasica*, *O. nasuta*, *O. orba*, *O. sangzhiensis*, *O. schmackeri*, *O. supranarina*, *O. swinhoana*, *O. tianmuii*, *O. tiannanensis*, *O. tormota*, *O. utsunomiyaorum*, *O. versabilis*, *O. yentuensis*, *O. yizhangensis*, and *O. yunnanensis*).

*Odorrana nagao* sp. nov. differs from other congeners by having a smaller body size in males (maximum SVL < 40.0 mm vs minimum SVL > 40.0 mm in *O. amamiensis*, *O. aureola*, *O. bacboensis*, *O. banaorum*, *O. bolavensis*, *O. cangyuanensis*, *O. chapaensis*, *O. chloronota*, *O. confusa*, *O. damingshanensis*, *O. dulongensis*, *O. exiliversabilis*, *O. geminata*, *O. grahami*, *O. graminea*, *O. hainanensis*, *O. hejiangensis*, *O. hosii*, *O. huanggangensis*, *O. ishikawae*, *O. indeprensa*, *O. jingdongensis*, *O. junlianensis*, *O. kuangwuensis*, *O. leporipes*, *O. lungshengensis*, *O. macrotympana*, *O. margaretae*, *O. mawphlangensis*, *O. mutschmanni*, *O. nanjiangensis*, *O. narina*, *O. nasica*, *O. nasuta*, *O. sangzhiensis*, *O. schmackeri*, *O. splendida*, *O. supranarina*, *O. swinhoana*, *O. tianmuii*, *O. tiannanensis*, *O. versabilis*, *O. wuchuanensis*, *O. yentuensis*, *O. yizhangensis*, and *O. yunnanensis*.

*Odorrana nagao* sp. nov. differs from other congeners by having a smaller body size in females (maximum SVL < 60.0 mm vs maximum SVL > 60.0 mm in *O. amamiensis*, *O. anlungensis*, *O. arunachalensis*, *O. aureola*, *O. bacboensis*, *O. banaorum*, *O. bolavensis*, *O. chapaensis*, *O. chloronota*, *O. confusa*, *O. damingshanensis*, *O. dulongensis*, *O. fengkaiensis*, *O. geminata*, *O. grahami*, *O. hainanensis*, *O. heatwolei*, *O. hejiangensis*, *O. hosii*, *O. huanggangensis*, *O. ichangensis*, *O. indeprensa*, *O. ishikawae*, *O. jingdongensis*, *O. junlianensis*, *O. kuangwuensis*, *O. kweichowensis*, *O. livida*, *O. lungshengensis*, *O. macrotympana*, *O. margaretae*, *O. monjerai*, *O. morafkai*, *O. mutschmanni*, *O. nanjiangensis*, *O. nasica*, *O. narina*, *O. nasuta*, *O. orba*, *O. sangzhiensis*, *O. schmackeri*, *O. splendida*, *O. sudianensis*, *O. supranarina*, *O. swinhoana*, *O. tiannanensis*, *O. versabilis*, *O. wuchuanensis*, *O. yentuensis*, *O. yizhangensis*, and *O. yunnanensis*.

## Discussion

Limestone karst ecosystems are considered ideal natural laboratories for studies in taxonomy, ecology, evolution, and zoogeography. Forests on limestone karst mountains contain a wide variety of microhabitats and are regarded as “terrestrial islands.” Consequently, the fauna inhabiting these areas often exhibits a high level of endemism (Clements et al. 2006). This new finding once again demonstrates the high diversity and local endemism of limestone ecosystems. The species description brings the total number of known species in the genus *Odorrana* from Vietnam to 26, and is the third species of the *O. lipuensis* group documented in Vietnam (Kilunda et al. 2025; Pham et al. 2025). This finding highlights the considerable diversity potential of the *Odorrana* genus in Vietnam, and warrants further studies to determine its actual species richness.

The new species, *Odorrana nagao*, is geographically closest to *O. liboensis* from Ba Be National Park, Thai Nguyen Province (ca. 74 km), yet their genetic divergences are 3.75% (gene 16S rRNA) and 9.49% (gene COI). In contrast, *Odorrana nagao* shows the lowest genetic divergence with *O. feii* (2.33% gene 16S rRNA and 6.33–6.52% gene COI), a species originally described from Guizhou, China (Li et al. 2025), with a much greater geographic distance of 556 km. Furthermore, *Odorrana nagao* differs genetically from *O. lipuensis* by 2.34% gene 16S rRNA and 7.48–7.67% gene COI, with a geographic distance of 172 km from Cao Bang Province (Pham et al. 2016a). The geographical distance between the two species of *Odorrana liboensis* and *O. lipuensis* recorded in Vietnam are ca 380–400 km from their type localities in China (Pham et al. 2016a, 2025). The complex distribution pattern of the *O. lipuensis* group suggests the potential for finding additional cryptic species of *Odorrana* in the border regions between China and Vietnam. Therefore, future studies with more extensive samplings are needed to enhance the understanding of the distribution pattern and geographic barriers of each species via sufficient distribution records and to elucidate species delimitations and speciation processes in concert with detailed studies on ecological adaptations.

## Supplementary Material

XML Treatment for Odorrana nagao

## Additional information

### Artificial Intelligence (AI) use

The authors accept full responsibility for the content of the manuscript, including the disclosure of any use of AI.

No AI tools were used in the preparation of this manuscript.

### Author ORCIDs

C.T. Pham https://orcid.org/0000-0001-5158-4526

C.V. Hoang https://orcid.org/0000-0002-0709-974X

M.H.T. Nguyen https://orcid.org/0009-0001-8322-010X

T.Q. Nguyen https://orcid.org/0000-0002-6601-0880

H.N. Ngo https://orcid.org/0000-0001-5471-965X

D.T. Le https://orcid.org/0000-0002-5925-7797

T. Ziegler https://orcid.org/0000-0002-4797-609X

### Data availability

All of the data that support the findings of this study are available in the main text or Supplementary Information.

## Supplementary materials

Supplementary material 1 GenBank accession numbers and associated samples used in this study Data type docx https://binary.pensoft.net/file/1654532This dataset is made available under the Open Database License (http://opendatacommons.org/licenses/odbl/1.0/). The Open Database License (ODbL) is a license agreement intended to allow users to freely share, modify, and use this Dataset while maintaining this same freedom for others, provided that the original source and author(s) are credited. Cuong The Pham, Chung Van Hoang, Mai Hong Thi Nguyen, Truong Quang Nguyen, Hai Ngoc Ngo, Dzung Trung Le, Thomas Ziegler

Supplementary material 2 References for morphological characters for congeners of the genus *Odorrana* Data type docx https://binary.pensoft.net/file/1654533This dataset is made available under the Open Database License (http://opendatacommons.org/licenses/odbl/1.0/). The Open Database License (ODbL) is a license agreement intended to allow users to freely share, modify, and use this Dataset while maintaining this same freedom for others, provided that the original source and author(s) are credited. Cuong The Pham, Chung Van Hoang, Mai Hong Thi Nguyen, Truong Quang Nguyen, Hai Ngoc Ngo, Dzung Trung Le, Thomas Ziegler

## References

[B1] Chan KO, Grismer LL (2022) GroupStruct: An R package for allometric size correction. Zootaxa 5124: 471–482. 10.11646/zootaxa.5124.4.4 35391110

[B2] Che J, Chen HM, Yang JX, Jin JQ, Jiang K, Yuan ZY, Murphy RW, Zhang YP (2012) Universal COI primers for DNA barcoding amphibians. Molecular Ecology Resources 12: 247–258. 10.1111/j.1755-0998.2011.03090.x 22145866

[B3] Clements R, Sodhi NS, Schilthuizen M, Ng PKL (2006) Limestone karsts of Southeast Asia: Imperiled arks of biodiversity. Bioscience 56: 733–742. 10.1641/0006-3568(2006)56[733:LKOSAI]2.0.CO;2

[B4] Darriba D, Taboada GL, Doallo R, Posada D (2012) jModelTest 2: more models, new heuristics and parallel computing. Nature Methods 9: 772. 10.1038/nmeth.2109 PMC4594756 22847109

[B5] Fei L, Hu SQ, Ye CY, Huang YZ (2009) Fauna Sinica. Amphibia (Vol. 2) Anura. Science Press, Beijing.

[B6] Fei L, Ye CY, Jiang JP (2012) Colored Atlas of Chinese Amphibians and Their Distributions. Sichuan Publishing Group, Sichuan Publishing House of Science & Technology. Sichuan, 619 pp.

[B7] Frost DR (2026) Amphibian Species of the World: an Online Reference. Version 6.2. American Museum of Natural History, New York, USA. https://amphibiansoftheworld.amnh.org/index.php [accessed on 15 March 2026]

[B8] Glaw F, Vences M (2007) A Field Guide to the Amphibians and Reptiles of Madagascar. 3^rd^ edn. FroschVerlag, Cologne, 496 pp.

[B9] Grismer L, Wood PL, Poyarkov NA, Le MD, Karunarathna S, Chomdej S, Suwannapoom C, Qi S, Liu S, Che J, Quah ESH, Kraus F, Oliver PM, Riyanto A, Pauwels OSG, Grismer JL (2021) Karstic landscapes are foci of species diversity in the World’s third-largest vertebrate genus *Cyrtodactylus* Gray, 1827 (Reptilia: Squamata; Gekkonidae). Diversity 13: 183. 10.3390/d13050183

[B10] Hoang DT, Chernomor O, von Haeseler A, Minh BQ, Vinh LS (2018) UFBoot2: Improving the ultrafast bootstrap approximation. Molecular Biology and Evolution 35: 518–522. 10.1093/molbev/msx281 PMC5850222 29077904

[B11] Kassambara A, Mundt F (2020) Factoextra: Extract and Visualize the Results of Multivariate Data Analyses. R Package Version 1.0.7. 10.32614/cran.package.factoextra [accessed 01 December 2025]

[B12] Kilunda FK, Yang SP, Nguyen LT, Le MV, Suwannapoom C, Stuart BL, Nguyen SN, Zuo AR, Zhang DC, Duan ZP, Duan PW, Yu ZB, Wu YH, Che J (2025) Unveiling hidden diversity in *Odorrana* (Anura, Ranidae) with description of a new species from Yingjiang, China and the first national records of *Odorrana heatwolei* in Thailand and Vietnam. Zoosystematics and Evolution 101: 2337–2367. 10.3897/zse.101.162366

[B13] Le S, Josse J, Husson F (2008) FactoMiner: A package for multivariate analysis. Journal of Statistical Software 25: 1–18. 10.18637/jss.v025.i01

[B14] Li S, Mu L, Jing J, Liu J, Cheng Y, Wuang B (2025) A new species of the genus *Odorrana* (Amphibia, Anura, Ranidae) from Guizhou Province, China. Zoosystematics and Evolution 101: 1949–1964. 10.3897/zse.101.161151

[B15] Lin SS, Li YH, Su HL, Yi H, Pan Z, Sun YJ, Zeng ZC, Wang J (2022) Discovery of a new limestone karst-restricted odorous frog from northern Guangdong, China (Anura, Ranidae, *Odorrana*). ZooKeys 1120: 47–66. 10.3897/zookeys.1120.87067 PMC9848673 36760328

[B16] Liu ZY, Wang YY (2014) The expansion of the distribution of *Odorrana wuchuanensis* and the reevaluation of its threatening category. Chinese Journal of Zoology 49: 766–771. 10.13859/j.cjz.201405017

[B17] Lleonart J, Salat J, Torres GJ (2000) Removing allometric effects of body size in morphological analysis. Journal of Theoretical Biology 205: 85–93. 10.1006/jtbi.2000.2043 10860702

[B18] Luo T, Wang S, Xiao N, Wang Y, Zhou J (2021) A new species of odorous frog genus *Odorrana* (Anura, Ranidae) from Southern Guizhou Province, China. Asian Herpetological Research 12: 381–398. 10.16373/j.cnki.ahr.200122

[B19] Mo Y, Chen W, Wu H, Zhang W, Zhou S (2015) A new species of *Odorrana* inhabiting complete darkness in a Karst Cave in Guangxi, China. Asian Herpetological Research 6: 11–17. 10.16373/j.cnki.ahr.140054

[B20] Nguyen LT, Schmidt HA, von Haeseler A, Minh BQ (2015) IQ-TREE: a fast and effective stochastic algorithm for estimating maximum-likelihood phylogenies. Molecular Biology and Evolution 32: 268–274. 10.1093/molbev/msu300 PMC4271533 25371430

[B21] Oksanen J, Blanchet FG, Friendly M, Kindt R, Legendre P, McGlinn D, Minchin PR, O’Hara RB, Simpson GL, Sólymos P, Stevens MHH, Szoecs E, Wagner H (2020) vegan: Community Ecology Package (version 2.5–7). 10.32614/cran.package.vegan [accessed 01 December 2025]

[B22] Pham CT, Nguyen TQ, Bernardes M, Nguyen TT, Ziegler T (2016a) First records of *Bufo gargarizans* Cantor, 1842 and *Odorrana lipuensis* Mo, Chen, Wu, Zhang et Zhou, 2015 (Anura: Bufonidae, Ranidae) from Vietnam. Russian Journal of Herpetology 23: 103–107.

[B23] Pham CT, Nguyen TQ, Le MD, Bonkowski M, Ziegler T (2016b) A new species of *Odorrana* (Amphibia: Anura: Ranidae) from Vietnam. Zootaxa 4084: 421–435. 10.11646/zootaxa.4084.3.7 27394273

[B24] Pham CT, Hoang CV, Ziegler T, Nguyen TQ (2025) First record of *Odorrana liboensis* Luo, Wang, Xiao, Wang & Zhou, 2021 (Amphibia: Anura: Ranidae) from Vietnam. Academia Journal of Biology 47(4): 1–10. 10.15625/2615-9023/23104

[B25] R Core Team (2025) R: A Language and Environment for Statistical Computing. R Foundation for Statistical Computing, Vienna, Austria. https://www.r-project.org

[B26] Rambaut A, Drummond AJ, Xie D, Baele G, Suchard MA (2018) Posterior summarisation in Bayesian phylogenetics using Tracer 1.7. Systematic Biology 67: 901–904. 10.1093/sysbio/syy032 PMC6101584 29718447

[B27] Ronquist FR, Teslenko T, Mark PVD, Ayres D, Darling A, Höhna S, Larget B, Liu L, Suchard MA, Huelsenbeck J (2012) MrBayes 3.2: Efficient Bayesian Phylogenetic inference and model choice across a large model space. Systematic Biology 61(3): 539–542. 10.1093/sysbio/sys029 PMC3329765 22357727

[B28] Simmons JE (2002) Herpetological collecting and collections management. Revised edition. Society for the Study of Amphibians and Reptiles. Herpetological Circular 31: 1–153.

[B29] Simon C, Frati F, Beckenbach A, Crespi B, Liu H, Flook P (1994) Evolution, weighting andphylogenetic utility of mitochondrial gene sequences and a compilation of conserved polymerase chain reaction primers. Annals of the Entomological Society of America 87(6): 651–701. 10.1093/aesa/87.6.651

[B30] Song HM, Qi S, Wang HT, Gong Y, Liu Y, Wang YY (2025) Definition and taxonomic revision of the karst-associated *Odorrana lipuensis* group (Anura, Ranidae), with a new species from Guangdong, China. Zoosystematics and Evolution 101: 935–952. 10.3897/zse.101.142746

[B31] Tamura K, Stecher G, Kumar S (2021) MEGA 11: Molecular Evolutionary Genetics Analysis Version 11. Molecular Biology and Evolution 38(7): 3022–3027. 10.1093/molbev/msab120 PMC8233496 33892491

[B32] Tanabe AS (2011) Kakusan4 and Aminosan: two programs for comparing nonpartitioned, proportional and separate models for combined molecular phylogenetic analyses of multilocus sequence data. Molecular Ecology Resources 11: 914–921. 10.1111/j.1755-0998.2011.03021.x 21592310

[B33] Thompson JD, Gibson TJ, Plewniak F, Jeanmougin F, Higgins DG (1997) The CLUSTAL_X windows interface: flexible strategies for multiple sequence alignment aided by quality analysis tools. Nucleic Acids Research 25: 4876–4882. 10.1093/nar/25.24.4876 PMC147148 9396791

[B34] Thorpe RS (1975) Quantitative handling of characters useful in snake systematics with particular reference to interspecific variation in the Ringed Snake *Natrix natrix* (L.). Biological Journal of the Linnean Society 7: 27–43. 10.1111/j.1095-8312.1975.tb00732.x

[B35] Thorpe RS (1983) A review of the numerical methods for recognizing and analyzing racial differentiation. In: Felsenstein J (Ed.) Numerical Taxonomy. NATo ASI Series (Series G: Ecological Sciences). Vol. 1. Springer, Berlin, 404–423. 10.1007/978-3-642-69024-2_43

[B36] Turan C (1999) A note on the examination of morphometric differentiation among fish populations: The Truss System. Turkish Journal of Zoology 23: 259–263.

