# Supplementary material for: A new species of Odorrana (Anura, Ranidae) from the limestone karst forest of northern Vietnam
Source: Zookeys. 2026 May 22;1280:245–63. doi: 10.3897/zookeys.1280.192981 (PMC13221660; doi:10.3897/zookeys.1280.192981)
Supplement: Supplementary material 1 — GenBank accession numbers and associated samples used in this study [file zookeys-1280-245_article-192981__-s001.docx]

**Supplementary material 1**. GenBank accession numbers and associated samples used in this study.

| **ID** | **Species** | **Locality** | **Voucher ID** | **GenBank accession No.** | | **Reference** |  |
| --- | --- | --- | --- | --- | --- | --- | --- |
|  |  |  |  | **16S rRNA** | **COI** |  |  |
| 1 | *Odorrana nagao* sp. nov. | Vietnam: Tuyen Quang: Cham Chu Nature Reserve | IB A.4505 | PZ332369 | PZ334602 | This study |  |
| 2 | *Odorrana nagao* sp. nov. | Vietnam: Tuyen Quang: Cham Chu Nature Reserve | IB A.4506 | PZ332370 | PZ334603 | This study |  |
| 3 | *Odorrana feii* | China: Guizhou: Xiuwen County | MT XW20250504001 | PV951782 | PV943472 | Li et al. 2025 |  |
| 4 | *Odorrana feii* | China: Guizhou: Xiuwen County | MT XW20250504002 | PV951783 | PV943473 | Li et al. 2025 |  |
| 5 | *Odorrana feii* | China: Guizhou: Xiuwen County | MT XW20250504003 | PV951784 | PV943474 | Li et al. 2025 |  |
| 6 | *Odorrana feii* | China: Guizhou: Jinsha County | MT JS20250504001 | PV951785 | PV943475 | Li et al. 2025 |  |
| 7 | *Odorrana feii* | China: Guizhou: Jinsha County | MT JS20250504002 | PV951786 | PV943476 | Li et al. 2025 |  |
| 8 | *Odorrana feii* | China: Guizhou: Jinsha County | MT JS20250504003 | PV951787 | PV943477 | Li et al. 2025 |  |
| 9 | *Odorrana lipuensis* | China: Guangxi: Yangshuo County: Yangshuo Town | SYS a004566 | PV347087 | PV341298 | Song et al. 2025 |  |
| 10 | *Odorrana lipuensis* | China: Guangxi: Yangshuo County: Yangshuo Town | SYS a004898 | PV347088 | PV341299 | Song et al. 2025 |  |
| 11 | *Odorrana liboensis* | China: Guizhou: Libo County: Maolan Nature Reserve | SYS a008240 | PV347080 | PV341295 | Song et al. 2025 |  |
| 12 | *Odorrana liboensis* | China: Guangxi: Daxin County: Xialei Town | SYS a003469 | PV347079 | PV341294 | Song et al. 2025 |  |
| 13 | *Odorrana liboensis* | China: Guangxi: Liuzhou City: Liunan District | SYS a008946 | PV347084 | PV341296 | Song et al. 2025 |  |
| 14 | *Odorrana liboensis* | China: Guangxi: Xincheng County: Masi Town | SYS a009224 | PV347085 | PV341297 | Song et al. 2025 |  |
| 15 | *Odorrana calciphila* | China: Guangdong: Yangshan County: Loushuiping Village | SYS a008923 | PV347065 | PV341279 | Song et al. 2025 |  |
| 16 | *Odorrana calciphila* | China: Guangdong: Yangshan County: Loushuiping Village | SYS a009287 | PV347066 | PV341280 | Song et al. 2025 |  |
| 17 | *Odorrana calciphila* | China: Guangdong: Yangshan County: Loushuiping Village | SYS a009288 | PV347067 | PV341281 | Song et al. 2025 |  |
| 18 | *Odorrana calciphila* | China: Guangdong: Yangshan County: Loushuiping Village | SYS a009289 | PV347068 | PV341282 | Song et al. 2025 |  |
| 19 | *Odorrana calciphila* | China: Guangdong: Yangshan County: Loushuiping Village | SYS a009290 | PV347069 | PV341283 | Song et al. 2025 |  |
| 20 | *Odorrana calciphila* | China: Guangdong: Ruyuan County: Datan River Nature Reserve | SYS a009295 | PV347070 | PV341284 | Song et al. 2025 |  |
| 21 | *Odorrana concelata* | China: Guangdong: Qingyuan City: Longlinchang Village | GEP a050 | OP137167 | / | Lin et al. 2022 |  |
| 22 | *Odorrana concelata* | China: Guangdong: Qingyuan City: Longlinchang Village | GEP a052 | OP137169 | / | Lin et al. 2022 |  |
| 23 | *Odorrana concelata* | China: Guangdong: Qingyuan City: Longlinchang Village | GEP a055 | OP137172 | / | Lin et al. 2022 |  |
| 24 | *Odorrana confusa* | China: Guangdong: Shixing County: Chebaling Nature Reserve | SYS a008595 | OR658982 | OR659591 | Song et al. 2023 |  |
| 25 | *Odorrana exiliversabilis* | China: Fujian: Wuyishan City: Mt. Wuyi | SYS a005931 | PV347072 | PV341286 | Song et al. 2025 |  |
| 26 | *Odorrana nasuta* | China: Hainan: Wuzhishan City: Mt. Wuzhi | SYS a005296 | PV347090 | PV344500 | Song et al. 2025 |  |
| 27 | *Odorrana versabilis* | China: Guangxi: Longsheng County: Huaping Nature Reserve | SYS a005118 | PV347094 | PV344501 | Song et al. 2025 |  |
| 28 | *Odorrana nasica* | China: Yunnan: Xichou County: Fadou Town | SYS a008842 | OR658988 | OR659609 | Song et al. 2023 |  |
| 29 | *Odorrana yentuensis* | China: Guangxi: Shangsi County: Mt. Shiwandashan | SYS a003543 | PV347095 | PV341305 | Song et al. 2025 |  |
| 30 | *Odorrana damingshanensis* | China: Guangxi: Nanning City: Mt. Daming | NNU 00690 | ON791420 | ON791393 | Chen et al. 2024 |  |
| 31 | *Odorrana tormota* | China: Anhui: Huangshan City: Mt. Huangshan | SYS a002699 | PV347093 | PV341303 | Song et al. 2025 |  |
| 32 | *Odorrana narina* | Japan: Okinawa: Okinawa Island | / | AB511287 | AB511288 | Kurabayashi et al. 2010 |  |
| 33 | *Odorrana chloronota* | China: Xizang: Medog County | SYS a008274 | PV347071 | PV341285 | Song et al. 2025 |  |
| 34 | *Odorrana graminea* | China: Hainan: Wuzhishan City: Mt. Wuzhi | SYS a005271 | ON391778 | ON381769 | Lyu et al. 2024 |  |
| 35 | *Odorrana leporipes* | China: Guangdong: Shaoguan City: Mt. Longtou | SYS a002769 | PV347078 | PV341293 | Song et al. 2025 |  |
| 36 | *Odorrana hosii* | Malaysia: Kuala Lumpur City | IABHU 21004 | AB511284 | AB511285 | Kurabayashi et al. 2010 |  |
| 37 | *Odorrana fengkaiensis* | China: Guangdong: Fengkai County: Heishiding Nature Reserve | SYS a002160 | KT315378 | OR659600 | Song et al. 2023 |  |
| 38 | *Odorrana hainanensis* | China: Hainan: Lingshui County: Mt. Diaoluo | SYS a007666 | PV347073 | PV341287 | Song et al. 2025 |  |
| 39 | *Odorrana hejiangensis* | China: Sichuan: Hejiang County: Zihuai Town | SYS a004936 | PV347074 | PV341288 | Song et al. 2023 |  |
| 40 | *Odorrana ichangensis* | China: Hubei: Changyang County: Gaojiayan Town | SYS a005475 | OR879765 | PV341289 | Li et al. 2024; Song et al. 2025 |  |
| 41 | *Odorrana kweichowensis* | China: Guangxi: Nanning City: Mt. Daming | SYS a005210 | PV347077 | PV341292 | Song et al. 2025 |  |
| 42 | *Odorrana schmackeri* | China: Hubei: Changyang County: Gaojiayan Town | SYS a005478 | PV347091 | PV341301 | Song et al. 2023 |  |
| 43 | *Odorrana huanggangensis* | China: Fujian: Wuyishan City: Mt. Wuyi | SYS a004148 | OR658951 | OR659603 | Song et al. 2023 |  |
| 44 | *Odorrana tianmuii* | China: Zhejiang: Hangzhou City: Mt. Tianmu | SYS a006431 | PV347092 | PV341302 | Song et al. 2025 |  |
| 45 | *Odorrana splendida* | Japan: Kagoshima: Amami Island | IABHU 5275 | AB511282 | AB511282 | Kurabayashi et al. 2010 |  |
| 46 | *Odorrana grahami* | China: Yunnan: Pingbian County: Mt. Dawei | SWFU 003918 | MW551527 | MW551527 | Wen et al. 2021 |  |
| 47 | *Odorrana jingdongensis* | China: Yunnan: Jingdong County: Xinmin Village | SYS a003922 | PV347075 | PV341290 | Song et al. 2023 |  |
| 48 | *Odorrana margaretae* | China: Sichuan: Emeishan City: Mt. Emei | SYS a005303 | ON391779 | ON381770 | Lyu et al. 2024 |  |
| 49 | *Odorrana kuangwuensis* | China: Sichuan: Nanjiang County: Mt. Guangwu | SYS a005409 | PV347076 | PV341291 | Song et al. 2025 |  |
| 50 | *Odorrana wuchuanensis* | China: Guangdong: Yangshan County: Jiangying Town | SYS a009109 | PP388290 | PV341304 | Song et al. 2024; Song et al. 2025 |  |
| 51 | *Odorrana lungshengensis* | China: Guangxi: Xing’an County: Mt. Maoer | SYS a002290 | PV347089 | PV341300 | Song et al. 2025 |  |
| 52 | *Odorrana yizhangensis* | China: Jiangxi: Jinggangshan City: Mt. Jinggang | SYS a004206 | PV347096 | PV341306 | Song et al. 2025 |  |
| 53 | *Odorrana anlungensis* | China: Guangxi: Tianlin County: Mt. Cenwanglaoshan | SYS a005974 | PV347064 | PV341278 | Song et al. 2025 |  |
| Outgroup | | | | | | | |
| 54 | *Amolops ricketti* | China: Fujian: Wuyishan City: Mt. Wuyi | SYS a004141 | MK263259 | MG991927 | Lyu et al. 2019 |  |
