# Supplementary material for: A new species of Odorrana (Anura, Ranidae) from the limestone karst forest of northern Vietnam
Source: Zookeys. 2026 May 22;1280:245–63. doi: 10.3897/zookeys.1280.192981 (PMC13221660; doi:10.3897/zookeys.1280.192981)
Supplement: Supplementary material 2 — References for morphological characters for congeners of the genus Odorrana [file zookeys-1280-245_article-192981__-s002.docx]

**Supplementary material 2.** References for morphological characters for congeners of the genus *Odorrana*.

| **No.** | **Species** | **Reference** |
| --- | --- | --- |
|  | *Odorrana absita* (Stuart & Chan-ard, 2005) | Stuart and Chan-Ard 2005 |
|  | *Odorrana amamiensis* (Matsui, 1994) | Matsui 1994 |
|  | *Odorrana andersonii* (Boulenger, 1882) | Bain et al. 2003 |
|  | *Odorrana anlungensis* (Liu & Hu, 1973) | Hu et al. 1973 |
|  | *Odorrana arunachalensis* Saikia, Sinha & Kharkongor, 2017 | Saikia et al. 2017 |
|  | *Odorrana aureola* Stuart, Chuaynkern, Chan-ard & Inger, 2006 | Stuart et al. 2006 |
|  | *Odorrana bacboensis* (Bain, Lathrop, Murphy, Orlov & Ho, 2003) | Bain et al. 2003 |
|  | *Odorrana banaorum* (Bain, Lathrop, Murphy, Orlov & Ho, 2003) | Bain et al. 2003 |
|  | *Odorrana bolavensis* (Stuart & Bain, 2005) | Stuart and Bain 2005 |
|  | *Odorrana calciphila* Song, Qi, Wang, Liu & Wang, 2025 | Song et al. 2025 |
|  | *Odorrana cangyuanensis* (Yang, 2008) | Yang and Rao 2008 |
|  | *Odorrana chapaensis* (Bourret, 1937) | Bain et al. 2009 |
|  | *Odorrana chloronota* (Günther, 1876) | Bain et al. 2003 |
|  | *Odorrana concelata* Wang, Zeng & Lin, 2022 | Lin et al. 2022; Song et al. 2025 |
|  | *Odorrana confusa* Song, Zhang, Qi, Lyu, Zeng & Wang, 2023 | Song et al. 2023 |
|  | *Odorrana damingshanensis* Chen, Mo, Lin & Qin, 2024 | Chen et al. 2024 |
|  | *Odorrana dulongensis* Liu, Che & Yuan, 2021 | Liu et al. 2021 |
|  | *Odorrana feii* Li, Mu, Jing, Liu, Cheng, and Wang, 2025 | Li et al. 2025 |
|  | *Odorrana exiliversabilis* Li, Ye & Fei, 2001 | Fei et al. 2001b |
|  | *Odorrana fengkaiensis* Wang, Lau, Yang, Chen, Liu, Pang & Liu, 2015 | Wang et al. 2015 |
|  | *Odorrana geminata* Bain, Stuart, Nguyen, Che & Rao, 2009 | Bain et al. 2009 |
|  | *Odorrana gigatympana* (Orlov, Ananjeva & Ho, 2006) | Orlov et al. 2006 |
|  | *Odorrana grahami* (Boulenger, 1917) | Boulenger 1917 |
|  | *Odorrana graminea* (Boulenger, 1900) | Boulenger 1900 |
|  | *Odorrana hainanensis* Fei, Ye & Li, 2001 | Fei et al. 2001a |
|  | *Odorrana heatwolei* (Stuart & Bain, 2005) | Stuart and Bain 2005 |
|  | *Odorrana hejiangensis* (Deng & Yu, 1992) | Deng and Yu 1992 |
|  | *Odorrana hosii* (Boulenger, 1891) | Boulenger 1920 |
|  | *Odorrana huanggangensis* Chen, Zhou & Zheng, 2010 | Chen et al. 2010b |
|  | *Odorrana ichangensis* Chen, 2020 | Shen et al. 2020 |
|  | *Odorrana indeprensa* (Bain & Stuart, 2006) | Bain and Stuart 2006 |
|  | *Odorrana ishikawae* (Stejneger, 1901) | Stejneger 1901; Kuramoto et al. 2011 |
|  | *Odorrana jingdongensis* Fei, Ye & Li, 2001 | Fei et al. 2001a |
|  | *Odorrana junlianensis* Huang, Fei & Ye, 2001 | Fei et al. 2009 |
|  | *Odorrana khalam* (Stuart, Orlov & Chan-ard, 2005) | Stuart et al. 2005 |
|  | *Odorrana kuangwuensis* (Liu & Hu, 1966) | Hu et al. 1966 |
|  | *Odorrana kweichowensis* Li, Xu, Lv, Jiang, Wei & Wang, 2018 | Li et al. 2018 |
|  | *Odorrana leishanensis* Li, Chen, Su, Liu, Tang & Wang, 2024 | Li et al. 2024 |
|  | *Odorrana leporipes* (Werner, 1930) | Bain et al. 2003 |
|  | *Odorrana liboensis* Luo, Wang, Xiao, Wang & Zhou, 2021 | Luo et al. 2021; Mo et al. 2022; Song et al. 2025 |
|  | *Odorrana lipuensis* Mo, Chen, Wu, Zhang & Zhou, 2015 | Mo et al. 2015; Pham et al. 2016a; Song et al. 2025 |
|  | *Odorrana livida* (Blyth, 1856) | Bain et al. 2003 |
|  | *Odorrana lungshengensis* (Liu & Hu, 1962) | Liu and Hu 1962 |
|  | *Odorrana macrotympana* (Yang, 2008) | Yang and Rao 2008 |
|  | *Odorrana margaretae* (Liu, 1950) | Liu 1950; Fei et al. 2009 |
|  | *Odorrana mawphlangensis* (Pillai & Chanda, 1977) | Pillai and Chanda 1977; Mahony 2008 |
|  | *Odorrana monjerai* (Matsui & Jaafar, 2006) | Matsui and Jaafar 2006 |
|  | *Odorrana morafkai* (Bain, Lathrop, Murphy, Orlov & Ho, 2003) | Bain et al. 2003 |
|  | *Odorrana mutschmanni* Pham, Nguyen, Le, Bonkowski & Ziegler, 2016 | Pham et al. 2016b |
|  | *Odorrana nanjiangensis* Fei, Ye, Xie & Jiang, 2007 | Fei et al. 2007b |
|  | *Odorrana narina* (Stejneger, 1901) | Matsui 1994 |
|  | *Odorrana nasica* (Boulenger, 1903) | Boulenger 1903 |
|  | *Odorrana nasuta* Li, Ye & Fei, 2001 | Fei et al. 2001b |
|  | *Odorrana orba* (Stuart & Bain, 2005) | Stuart and Bain 2005 |
|  | *Odorrana sangzhiensis* Zhang, Li, Hu & Yang, 2021 | Zhang et al. 2021 |
|  | *Odorrana schmackeri* (Boettger, 1892) | Shen et al. 2020 |
|  | *Odorrana sinica* (Ahl, 1927) | Bain et al. 2003 |
|  | *Odorrana splendida* Kuramoto, Satou, Oumi, Kurabayashi & Sumida, 2011 | Kuramoto et al. 2011 |
|  | *Odorrana sudianensis* Kilunda, Yu, Wu, and Che, 2025 | Kilunda et al. 2025 |
|  | *Odorrana supranarina* (Matsui, 1994) | Matsui 1994 |
|  | *Odorrana swinhoana* (Boulenger, 1903) | Boulenger 1903 |
|  | *Odorrana tianmuii* Chen, Zhou & Zheng, 2010 | Chen et al. 2010a |
|  | *Odorrana tiannanensis* (Yang & Li, 1980) | Yang and Li 1980 |
|  | *Odorrana tormota* (Wu, 1977) | Herpetological Department, Sichuan Biological Research Institute 1977 |
|  | *Odorrana utsunomiyaorum* (Matsui, 1994) | Matsui 1994 |
|  | *Odorrana versabilis* (Liu & Hu, 1962) | Liu and Hu 1962 |
|  | *Odorrana wuchuanensis* (Xu, 1983) | Wu et al. 1983 |
|  | *Odorrana yentuensis* Tran, Orlov & Nguyen, 2008 | Tran et al. 2008 |
|  | *Odorrana yizhangensis* Fei, Ye & Jiang, 2007 | Fei et al. 2007a |
|  | *Odorrana yunnanensis* Anderson, 1879 | Fei et al. 2009 |
